# Functional and transcriptomic characterization of cisplatin-resistant AGS and MKN-28 gastric cancer cell lines

**DOI:** 10.1371/journal.pone.0228331

**Published:** 2020-01-28

**Authors:** Barbara Mora-Lagos, Irene Cartas-Espinel, Ismael Riquelme, Alyssa C. Parker, Stephen R. Piccolo, Tamara Viscarra, María Elena Reyes, Louise Zanella, Kurt Buchegger, Carmen Ili, Priscilla Brebi

**Affiliations:** 1 Laboratory of Integrative Biology (LIBi), Scientific and Technological Bioresource Nucleus- Center for Excellence in Translational Medicine (BIOREN-CEMT), Universidad de La Frontera, Temuco, Chile; 2 Dirección de Investigación, Vicerrectoría de Investigación y Postgrado, Universidad Autónoma de Chile, Temuco, Chile; 3 Instituto de Ciencias Biomédicas, Facultad de Ciencias de la Salud, Universidad Autónoma de Chile, Temuco, Chile; 4 Department of Biology, Brigham Young University, Provo, Utah, United States of America; 5 Department of Basic Sciences, School of Medicine, Universidad de La Frontera, Temuco, Chile; The University of Hong Kong, HONG KONG

## Abstract

Gastric cancer (GC) is a significant cancer-related cause of death worldwide. The most used chemotherapeutic regimen in GC is based on platinum drugs such as cisplatin (CDDP). However, CDDP resistance reduces advanced GC survival. *In vitro* drug-resistant cell model would help in the understanding of molecular mechanisms underlying this drug-resistance phenomenon. The aim of this study was to characterize new models of CDDP-resistant GC cell lines (AGS R-CDDP and MKN-28 R-CDDP) obtained through a stepwise increasing drug doses method, in order to understand the molecular mechanisms underlying chemoresistance as well as identify new therapeutic targets for the treatment of GC. Cell viability assays, cell death assays and the expression of resistance molecular markers confirmed that AGS R-CDDP and MKN-28 R-CDDP are reliable CDDP-resistant models. RNA-seq and bioinformatics analyses identified a total of 189 DEGs, including 178 up-regulated genes and 11 down-regulated genes, associated mainly to molecular functions involved in CDDP-resistance. DEGs were enriched in 23 metabolic pathways, among which the most enriched was the *inflammation mediated by chemokine and cytokine signaling pathway*. Finally, the higher mRNA expression of *SERPINA1*, *BTC* and *CCL5*, three up-regulated DEGs associated to CDDP resistance found by RNA-seq analysis was confirmed. In summary, this study showed that AGS R-CDDP and MKN-28 R-CDDP are reliable models of CDDP resistance because resemble many of resistant phenotype in GC, being also useful to assess potential therapeutic targets for the treatment of gastric cancers resistant to CDDP. In addition, we identified several DEGs associated with molecular functions such as binding, catalytic activity, transcription regulator activity and transporter activity, as well as signaling pathways associated with inflammation process, which could be involved in the development of CDDP resistance in GC. Further studies are necessary to clarify the role of inflammatory processes in GC resistant to CDDP and these models could be useful for these purposes.

## Introduction

Gastric cancer (GC) is the sixth most common malignancy and the third leading cause of cancer-related deaths in both sexes in the world, making it a major global public health problem [1]. As most patients are diagnosed in advanced or metastatic stages, surgery is not always appropriate [2], and chemotherapy becomes the only strategy to improve survival rates and mitigate adverse symptoms [3]. In this regard, cisplatin (CDDP) is still one of the most used drugs in first-line therapy against advanced GC [4,5]. The platinum compound CDDP can bind covalently to DNA, forming adducts that inhibit DNA replication subsequently causing transcription inhibition, cell-cycle arrest, DNA repair deficiency and apoptosis [6]. Unfortunately, the overall 5-year survival rate for GC patients who received chemotherapy and perioperative surgery remains below 37%, while in patients with no resectable tumors, chemotherapy has shown scarce benefits with an average survival of ~10 months due to reduced treatment efficiency, resulting in tumor regrowth and lower survival [7]. Therefore, chemoresistance—whether intrinsic or acquired—is a multifactorial phenomenon that represents the most important cause of treatment failure and mortality in GC [8]. Drug resistance is caused by several processes at different levels that can act independently or in combination [9]. Different experimental studies have led to the identification of diverse molecular mechanisms involved in chemoresistance including drug inactivation, alteration of drug flux, inactivation of death signaling pathways, changes in drug metabolism and epigenetics, gene mutation or amplification in drug targets and enhanced DNA repair mechanisms [10–12].

The development of more reliable *in vitro* models of acquired or induced drug resistance is a useful approach to better understand the mechanisms that trigger clinical resistance to chemotherapeutics. In addition, *in vitro* models can clarify the cellular and molecular mechanisms of novel anticancer agents, enabling comparisons with parental cells and intrinsically resistant cells [13]. The aim of this study was to characterize functionally models of CDDP-resistant gastric cancer based on two gastric cancer cell lines (AGS and MKN-28), which were developed through administering stepwise increases in drug dose.

## Materials and methods

### Ethics statements

This study was approved by Ethical Committee of Universidad de La Frontera (Approval certificate N°83/2015).

### Drugs

Cisplatin (CDDP) was purchased from Selleck Chemicals (SelleckChem, USA). CDDP was reconstituted at a concentration of 3.3 mM diluted in 0.9% (p/v) NaCl and aliquots of stock solution were stored at −80°C.

### Cell lines and culture conditions

AGS and MKN-28 cell lines were generously provided by Dr. Richard Peek (Vanderbilt University, Nashville, USA). AGS was established from a gastric adenocarcinoma obtained from a 54-year-old female [14] and MKN-28 from a moderately differentiated gastric tubular adenocarcinoma obtained from a 70-year-old female [15]. AGS and MKN-28 were cultured in RPMI-1640 medium supplemented with 10% (v/v) fetal bovine serum (Thermofischer, USA) and 1% (v/v) penicillin and streptomycin (Thermofischer, USA). Cells were maintained at 37°C in a 95% humidified atmosphere and 5% CO_2_ conditions. Cells were subcultured at 80% confluence and harvested after treatment with 0.25% trypsin and 0.02% EDTA (Corning, USA).

### Development of CDDP-resistant cell lines

Induced drug-resistant cell lines, CDDP-resistant AGS cells (AGS R-CDDP) and CDDP-resistant MKN-28 cells (MKN-28 R-CDDP) were developed following Coley’s protocol [16]. Briefly, the drug sensitivity of the parental cells was tested by establishing the starting dose of treatment at 20% of the EC_50_ concentration. Cells were seeded according to doubling time, and the starting dose of the drug was incorporated into the cells when they presented 20% confluence. The increase in drug doses was made every two subcultures, by doubling each previous concentration. The cycle was repeated 30 times. Once cells acquired cisplatin resistance they were grown in drug-free medium for one month, frozen in liquid nitrogen and then awakened in medium containing CDDP to confirm the level of drug resistance. The time for the development of this drug-resistant model was 12 months.

### Drug sensitivity assay

Drug sensitivity analyses were performed using a standard viability assay (MTT assay). Briefly, cells were seeded in 96-well plates (4x10^3^ for parental cells and 5.5x10^3^ for resistant cells according to their doubling time) in 100 μL of culture medium and incubated for 24 H to allow cell attachment and to reach a 50% confluence. Next, cells were exposed for 72 H at different concentrations of CDDP, ranging from 0.01 μM to 1000 μM. Cells without CDDP were used as controls. After 72 H of incubation the medium was removed, and cells were washed with 100 μL of DPBS/Modified (Thermofischer, USA). Then, 0.5 mg/mL of MTT was added to each well, followed by 2 H incubation. As only functional mitochondrial dehydrogenase enzymes from viable cells can reduce MTT to form formazan, 100 μL of propanol was used to fully dissolve this purple precipitate. Absorbance was measured at 570 nm using the Infinite^®^ NanoQuant spectrophotometer (TECAN, Switzerland). The EC_50_ values (drug concentration that inhibited cell growth at 50%) were estimated through the dose-response curve after 72 H of incubation under different drug concentrations. In this case, the percentage of viable cells was plotted according to the corresponding drug concentrations, obtaining the values of half maximal effective concentration (EC_50_) by non-linear regression. The resistance index (RI) values were calculated by dividing the EC_50_ values of resistant cell lines by the EC_50_ values of parental cell lines, defining arbitrarily the chemoresistance as RI ≥2.

### Cell death assay

A cell death assay was performed using the Dead Cell Apoptosis Kit with Alexa Fluor^™^ 488 annexin V and propidium iodide (PI) (Invitrogen) according to the manufacturer’s instructions. Briefly, parental and resistant cells were seeded in 6-well plates (5x10^4^ parental cells and 7x10^4^ resistant cells, according to doubling time) in 2 mL of culture medium and incubated for 24 H to allow cell attachment. Then, cells were treated for 72 H at EC_50_ concentrations of resistant cells (26.05 μM of CDDP for parental AGS cells and AGS R-CDDP cells; 33.61 μM of CDDP for parental MKN-28 cells and MKN-28 R-CDDP cells). Following incubation, cells were harvested by trypsin and the pellets were centrifuged, washed with 1X PBS and resuspended in 100 μL of 1X annexin buffer. For staining, cells were incubated with 5 μL of Alexa Fluor® 488 annexin V and 1 μL of 100 μg/mL Propidium iodine (PI) at 37°C for 15 minutes. Finally, cells were resuspended with 400 μL of 1X annexin buffer and collected for analysis by flow cytometry (FACSCANTO II, BD, USA). 5% of DMSO was used as an apoptosis-inducing agent. Early apoptotic cells (annexin V-positive, PI-negative), late apoptotic cells (annexin V-positive and PI-positive) and annexin V-negative and PI-positive cell populations were all considered dead cells. The fluorescence was read at maximum excitation/emission of 499⁄521 for Alexa Fluor® 488 annexin V and 535⁄617 for PI.

### RNA extraction and real time-PCR analysis

The expression profile of molecular markers involved in CDDP resistance (*ABCC2*, *ATP7A* and *CTR1* genes), as well as the expression profile of genes obtained by subsequent RNA-seq analysis (*SERPINA1*, *BTC* and *CCL5* genes) were performed by qRT-PCR. Total RNA was extracted from ~2.0 x10^6^ cells using TRIzol Reagent (Thermofischer, USA) according to the manufacturer’s instructions. RNA concentration and integrity were evaluated at 260 nm using the Infinite®NanoQuant spectrophotometer (TECAN, Switzerland) and by gel electrophoresis. Then, RNA was treated with DNase I (Promega Corp, USA) and first-strand cDNA was prepared from 2 μg of RNA in a total reaction volume of 20 μL using M-MLV reverse transcriptase 200 U/μL (Promega Corp, USA) at 42°C for 60 min. Subsequently, cDNA was amplified by qPCR using Brilliant II Ultra-Fast SYBR® Green qPCR Master Mix according to the manufacturer’s protocol in the Stratagene Mx-3000p real-time PCR system (Agilent Technologies, USA). Relative expression was determined using the 2^-ΔΔCT^ method, using *ACTB* as the reference gene. Sequences of oligonucleotides used in this study are detailed in Table 1.

**Table 1:** Sequences of oligonucleotides used in this study.

Gene	Primer sequence 5'-3'	Amplicon (bp)
*ABCC2*	Fw CACAGTCCCTGCTGTTCGAT	120
	Rv AGGGACAGGAACCAGGAGTT	
*ATP7A*	Fw AAACTGCAAGGTGTTCAGCG	118
	Rv AGCCCATAGCTTCAATCTGCT	
*CTR1*	Fw ACCATCACCCAACCACTTCA	115
	Rv CCGGAAAACAGTAGTTCCACA	
*SERPINA1*	Fw CAGGATCACCCAACCTTCAACA	191
	Rv TTCAGGCCCTCCAGGATTTCAT	
*BTC*	Fw CTGCAAAGTGCCTTGCTCAT	192
	Rv TGTTGCTACCTAACCAGTTGCT	
*CCL5*	Fw TGGGTTCGGGAGTACATCAA	162
	Rv GTAGAATCTGGGCCCTTCAA	
*ACTB*	Fw ATCATTGCTCCTCCTGAGC	107
	Rv ACTCCTGCTTGCTGATCCAC	

### RNA-seq analysis

Total RNA was isolated from ~2.0 x10^6^ AGS cells and ~1.0 x10^6^ MKN-28 cells using TRIzol Reagent (Thermofischer, USA) according to the manufacturer’s instructions. RNA integrity was checked by Agilent Bioanalyzer 2100 with a RNA Integrity Number (RIN) value ≥ 7.0. cDNA libraries were prepared according to KAPA Stranded RNA-Seq Kit (KAPA Biosystems, USA) with the RiboErase system for ribosomal RNA depletion. Finally, the library´s quality and quantity were analyzed using the Agilent Bioanalyzer 2100 and Life Technologies Qubit3.0 Fluorometer, respectively. 150 bp paired-end sequencing was performed on an Illumina HiSeq 4000 (Illumina Inc., USA) instrument. The quality control (QC) of raw reads was checked with the FastQC tool [17]. RNA-seq data from the parental and CDDP-resistant cell lines were analyzed using a series of bioinformatics tools and Python (version 3.6.5), R (version 3.3.2), and Bash (version 4.1.2) scripts [18–20]. First, the RNA-seq reads were trimmed with the Trimmomatic tool (version 0.38) to discard low-quality reads, remove adaptor sequences, and eliminate poor-quality bases [21]. Then, the reads were aligned to a reference genome using the sequence alignment tool Kallisto (version 0.44.0) [22]. The human reference genome used was Release 28 from The ENCODE Project [23]. After reads alignment, a differential expression analysis was performed between the CDDP-resistant cell lines group and the parental cell lines group using the Sleuth tool (version 0.30.0) [24]. A P-value <0.05 and fold change >2 were used as a cutoff to choose the differential expressed genes (DEGs). The scripts used for this analysis can be found at https://osf.io/ck39n/. Two biological replicates per cell line type were evaluated by RNA-seq analysis. Overexpressed genes were validated using qPCR (as described before) and western blot analyses comparing parental and CDDP-resistant cell lines. The sequencing data were submitted to the National Center for Biotechnology Information (NCBI) Sequence Read Archive (SRA) under accession number PRJNA591481.

### Gene ontology annotation and signaling pathway analysis

Gene ontology (GO) annotation and signaling pathway analysis of DEGs between CDDP-resistant and parental cell lines, were analyzed using the PANTHER Classification System (version 14.0) [25] and Metascape tool [26].

### Western blot analysis

Parental and resistant cellular pellet was obtained at 70% of confluence after three subcultures. Subsequently, protein lysates were harvested using RIPA buffer (50 mM Tris, pH 7.2; 150 mM NaCl; 1% Triton X-100; and 0.1% SDS) containing protease (1:100, Roche, USA) and phosphatase (1:100, Sigma-Aldrich, USA) inhibitors. The protein concentrations were determined using a bicinchoninic acid assay (Pierce, Thermo Scientific, USA). An amount of 40 μg of the proteins were separated by SDS-PAGE and transferred (Bio-Rad, USA) to PVDF membranes (Millipore, USA). Human SERPINA1, BTC and CCL5 protein expression levels were quantified using goat monoclonal antibody specific for human SERPINA1 and BTC (1:500, Santa Cruz Biotechnology, USA) and goat polyclonal antibody specific for CCL5 protein (1:500, R&D Systems, USA). The expression levels of these three proteins were standardized to human β-actin using a rabbit monoclonal anti- β-actin antibody (1:5000, Cell Signaling, USA). Primary antibodies were detected using donkey anti-goat or goat anti-mouse or mouse anti-rabbit- radish peroxidase (HRP)-conjugated secondary antibodies (1:5000, Santa Cruz Biotechnology, USA). Immunoreactive bands were visualized using myECL^™^ Imager (Thermo Scientific, USA) according to the manufacturer’s instructions, and then quantified by densitometry using a ChemiGenius Gel Bio Imaging System (Syngene, USA).

### Statistical analysis

All the experiments were performed in biological and technical triplicates for each condition. Data were analyzed using the GraphPad Prism 5.0 software (GraphPad, USA). Cell viability, cell death assay, qPCR and protein levels data were analyzed using the Mann-Whitney test. The EC_50_ values were calculated from dose-response curves using non-linear regression. P<0.05 values were considered statistically significant with 95% confidence interval.

## Results

### Sensitivity to CDDP in parental and CDDP-resistant GC cell lines

**Fig 1:**
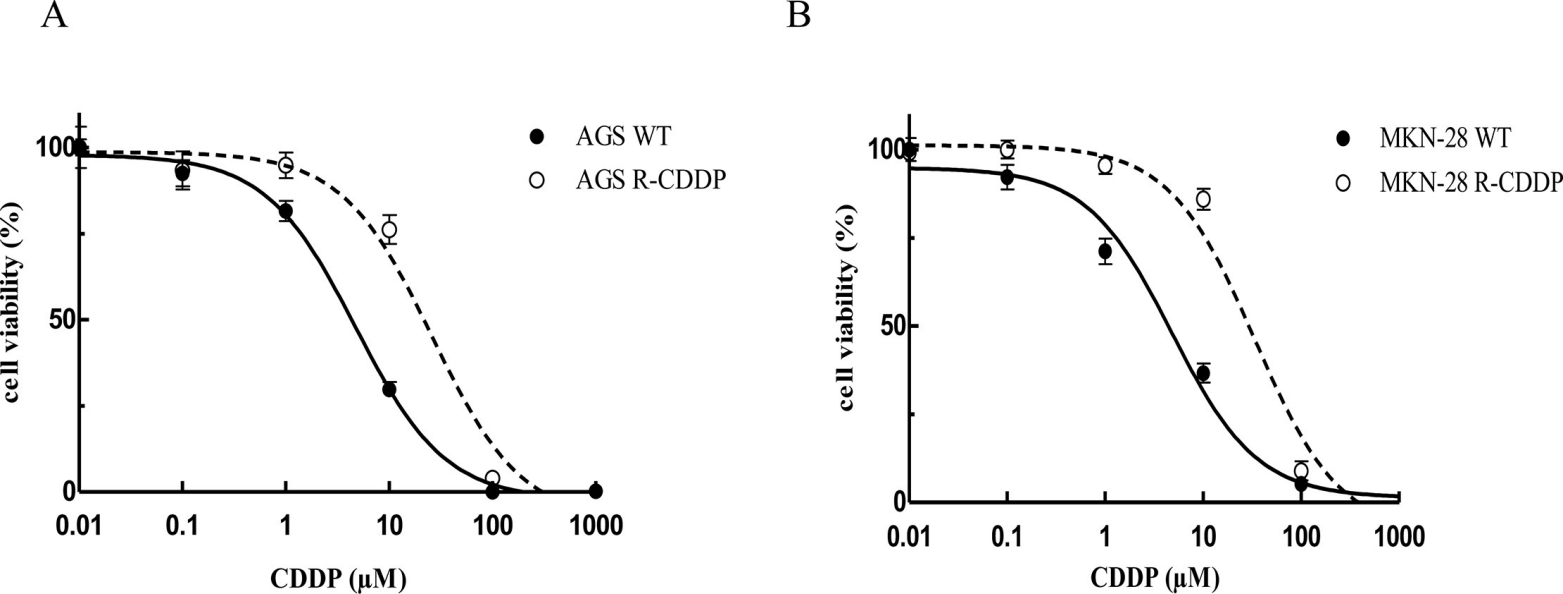
Drug sensitivity of resistant and parental cell lines by MTT assay. (A) Dose-response curves for cisplatin-resistant AGS cells (AGS R-CDDP) and parental AGS cells (AGS WT); (B) Dose-response curves for cisplatin-resistant MKN-28 cells (MKN-28 R-CDDP) and parental MKN-28 cells (MKN-28 WT). Data were expressed as mean ± SD of three biological replicates.

Viability assays made it possible to obtain dose-response curves (Fig 1) and non-linear regression determined EC_50_ values. The degree to which CDDP induced resistance was estimated as the ratio of the EC_50_ of each resistant line with its respective parental cell lines. EC_50_ values and RI are summarized in Table 2. Results show that CDDP-resistant cell lines presented a significant tolerance to higher concentrations of CDDP, increasing 5.4-fold for AGS R-CDDP and 6.8-fold for MKN-28 R-CDDP compared to their respective parental cell line.

**Table 2:** Values of EC_50_ and resistance index in parental and CDDP-resistant cells.

Parental cell lines	EC_50_ (μM)	Resistant cell lines	EC_50_ (μM)	RI
AGS WT	4.84±0.77	AGS R-CDDP	26.05±0.24	5.4
MKN-28 WT	4.95±1.95	MKN-28 R-CDDP	33.61±1.02	6.8

EC: Effective concentration (Drug concentration in μM that inhibited cell growth by 50%). RI: Resistant index; WT: Wild type or parental cell line; CDDP: Cisplatin.

### Response to CDDP‐induced cell death in both parental and CDDP-resistant GC cell lines

**Fig 2:**
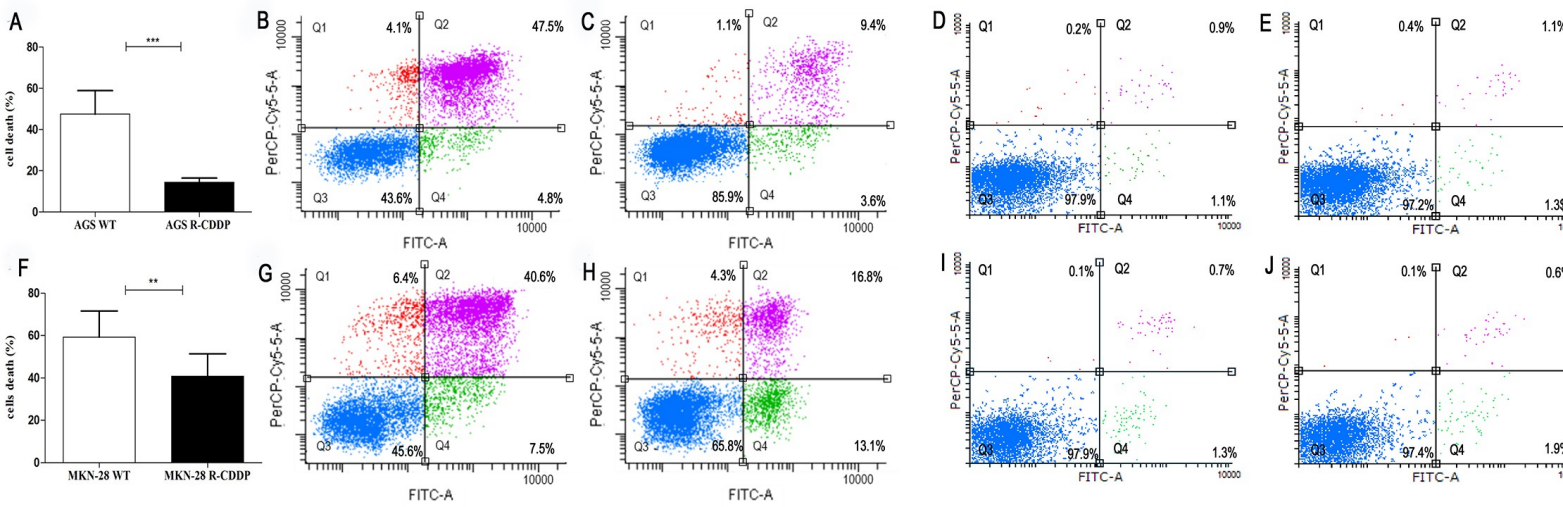
Cell death analysis through flow cytometry with annexin-V and PI) staining of AGS and MKN28 cells treated with cisplatin. (A) Percentage of death parental AGS cells (AGS WT) compared with CDDP-resistant AGS cells (AGS R-CDDP). This figure includes annexin V-positive/PI-negative cells at Q4 coordinate, annexin V/PI double positive cells at Q2, and annexin V-negative/PI-positive cells at Q1; (B) Representative dot plots of AGS WT treated with 26.05 μM of CDDP; (C) Representative dot plots of AGS R-CDDP treated with 26.05 μM of CDDP; (D) Representative dot plots of AGS WT control (without incubation with CDDP); (E) Representative dot plots of AGS R-CDDP control (without incubation with CDDP); (F) Percentage of death parental MKN-28 cells (MKN-28 WT) compared with CDDP-resistant MKN-28 cells (MKN-28 R-CDDP). This figure includes annexin V-positive/PI-negative cells at Q4 coordinate, annexin V/PI double positive cells at Q2, and annexin V-negative/PI-positive at Q1; (G) Representative dot plot of MKN-28 WT treated with 33.61 μM of CDDP; (H) Representative dot plot of MKN-28 R-CDDP treated with 33.61 μM of CDDP; (I) Representative dot plots of MKN-28 WT control (without incubation with CDDP); (J) Representative dot plots of MKN-28 R-CDDP control (without incubation with CDDP). The cells represented in coordinates Q4, Q2 and Q1 were all considered as dead cells. Mann-Whitney test was used to compare groups. *P<0.05, **P<0.01 and ***P<0.001. Data were expressed as mean ± SD of three biological replicates.

Analysis of cell death by flow cytometry using annexin V and PI staining showed significantly lower cell death rates in CDDP-resistant cells than parental cell lines. When overall death percentages (annexin V-positive/PI-negative; Q4, annexin V/PI double positive cells; Q2, and annexin V-negative/PI-positive; Q1) were compared, AGS R-CDDP cells showed a statistically significant reduced cell death percentage (14.5%) compared with parental AGS cells (47.5%) (P<0.001; Fig 2A). Representative cytometric profiles according to cell death (dot plots) showed an extensive cell death and a decrease of live cell population in AGS cells (Fig 2B) compared with AGS R-CDDP cells (Fig 2C). Representative dot plots of AGS WT and AGS R-CDDP controls (without incubation with CDDP) are shown in Fig 2D and Fig 2E, respectively.

Similarly, MKN-28 R-CDDP cells showed a statistically lower cell death percentage (overall death percentages; 40.8%) than parental MKN-28 cells (overall death percentages; 59.2%) (P<0.01; Fig 2F). Representative cytometric profiles according to cell death (dot plots) showed an extensive cell death and a decrease of live cell population in parental MKN-28 cells (Fig 2G) compared with MKN-28 R-CCDP cells (Fig 2H). Representative dot plots of MKN-28 WT and MKN-28 R-CDDP controls (without incubation with CDDP) are shown in Fig 2I and Fig 2J, respectively.

### Relative expression of molecular markers involved in CDDP resistance

**Fig 3:**
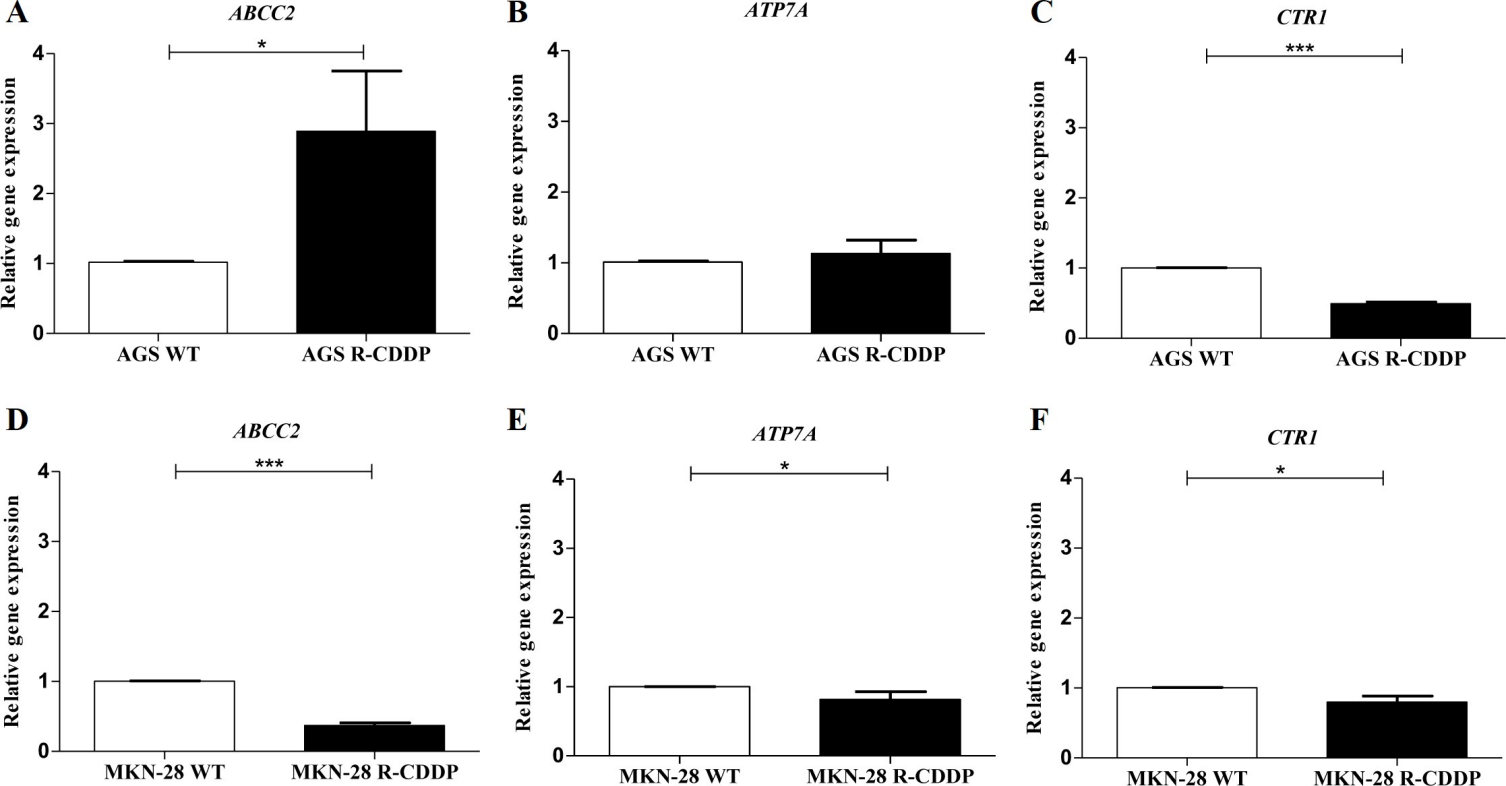
Relative expression of molecular markers involved in cisplatin resistance. (A) *ABCC2* gene expression in AGS WT cells and AGS R-CDDP cells; (B) *ATP7A* gene expression in AGS WT and AGS R-CDDP cells; (C) *CTR1* gene expression in AGS WT and AGS R-CDDP cells; (D) *ABCC2* gene expression in MKN-28 WT cells and MKN-28 R-CDDP cells; (E) *ATP7A* gene expression in MKN-28 WT and MKN-28 R-CDDP cells; (F) *CTR1* gene expression in MKN-28 WT and MKN-28 R-CDDP cells. The mRNA expression was evaluated by qRT-PCR using 2^-ΔΔCT^ method with *ACTB* gene as the control. Mann-Whitney test was used to compare groups. Values of P<0.05 were considered statistically significant. *P<0.05, **P<0.01 and ***P<0.001. Data were expressed as mean ± SD of three biological replicates.

Gene expression of targets associated with CDDP resistance such as *ABCC2*, *ATP7A* and *CTR1* were evaluated on the AGS and MKN-28 cell lines subjected to both conditions: parental and CDDP-resistant cells (Fig 3). In AGS cells, a significant increase in *ABCC2* expression was observed in AGS R-CDDP compared to parental AGS cells (P<0.05; Fig 3A). *ATP7A* expression in AGS R-CDDP also showed a mild increase compared to parental AGS cells (Fig 3B), but this difference was not significant. On the other hand, expression of *CTR1* was significantly reduced in AGS R-CDDP (P<0.001; Fig 3C) compared to parental AGS cells. In MKN-28 cells, a significant decrease in the expression of *ABCC2* (P<0.001; Fig 3D), *ATP7A* (P<0.05; Fig 3E) and *CTR1* (P<0.05; Fig 3F) was shown in MKN-28 R-CDDP cells compared to parental MKN-28 cells.

### Transcriptomic sequencing analysis in parental and CDDP-resistant GC cell lines

**Fig 4:**
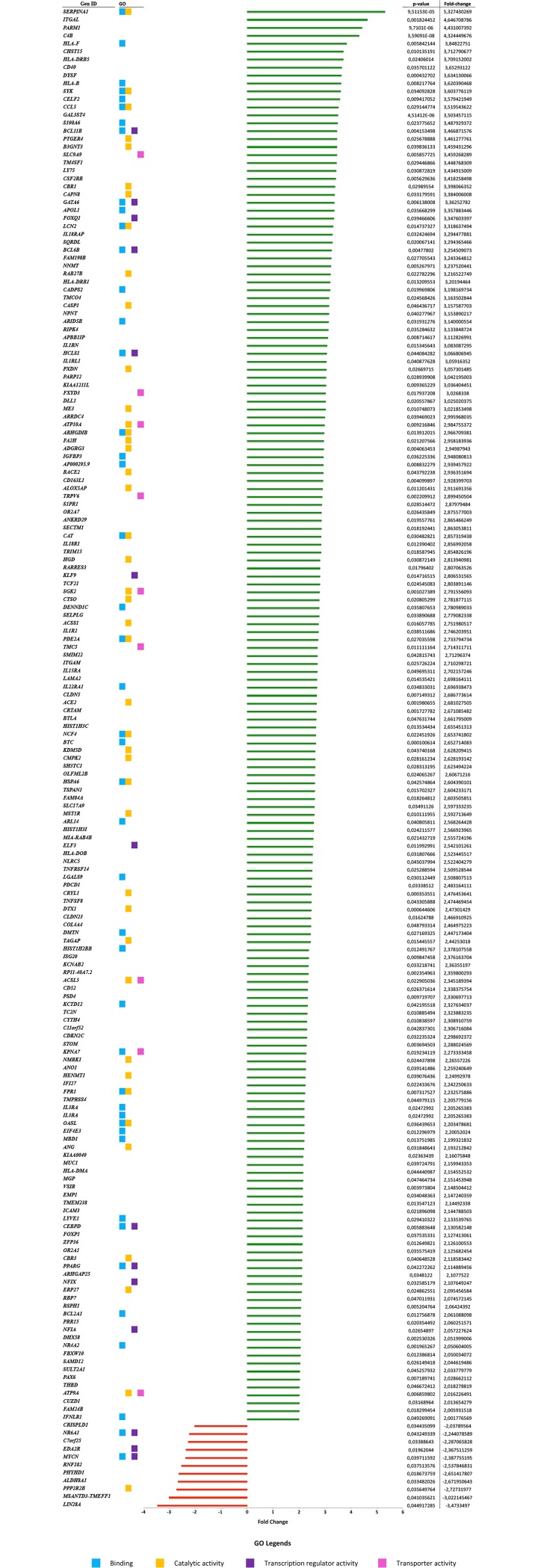
Differentially expressed genes (DEGs) in CDDP-resistant cell lines AGS R-CDDP and MKN-28 R-CDDP. Up-regulated (green bars) and down-regulated (red bars) genes are ordered according to fold-change value. Gene ontology (GO) is shown according to the four main categories related to CDDP-resistant phenotype obtained for molecular functions: *binding* (blue dots), *catalytic activity* (orange dots), *transcription regulator activity* (purple dots) and *transporter activity* (pink dots). The adjacent table shows P-values and fold-change values for each gene.

An RNA-seq approach was used to assess the differential expression across the transcriptome in CDDP-resistant gastric cancer cells (AGS R-CDDP and MKN-28 R-CDDP) and their parental cells (AGS and MKN-28 cells without drug exposure). A total of 189 genes were found significantly differentially expressed (P<0.05 and fold change ≥2); 178 genes were up-regulated, and 11 genes were found down-regulated in CDDP-resistant cells compared to parental cells (Fig 4).

### Gene ontology analysis

**Fig 5:**
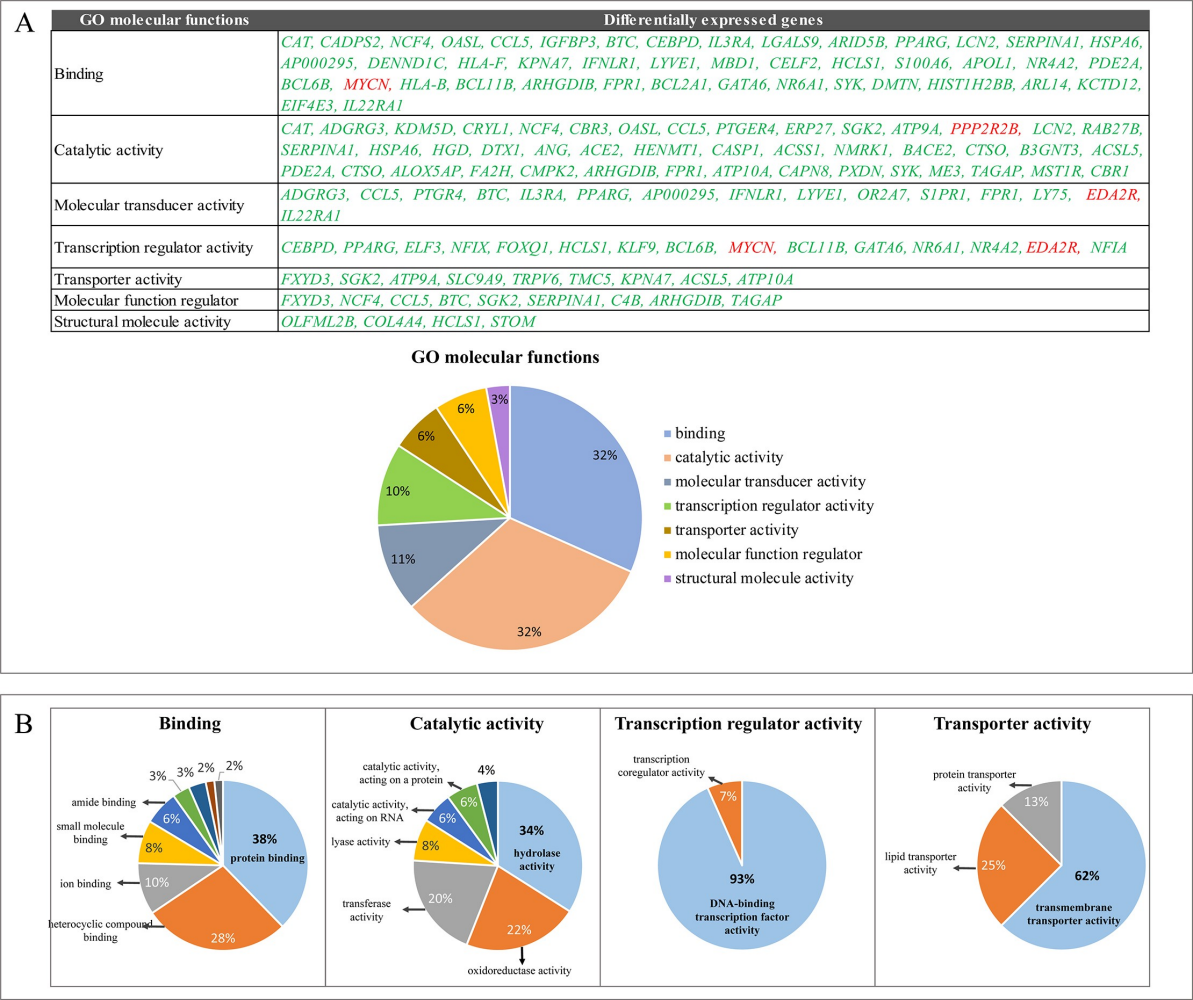
Gene ontology (GO) analysis of the differential expressed genes (DEGs) using PANTHER classification system. (A) The categories of molecular functions of DEGs. Up-regulated genes are shown in green and down-regulated genes are shown in red. (B) The main molecular functions related to the CDDP-resistant phenotype and their corresponding subcategories.

Using the PANTHER Classification System, functional analysis of the DEGs between the CDDP-resistant cells group and parental cells group was performed using GO annotation. Of the 189 DEGs, 139 were enriched into 7 molecular functions: *binding* (32%), *catalytic activity* (32%), *molecular transduces activity* (11%), *transcription regulator activity* (10%), *transporter activity* (6%), *molecular function regulator* (6%) and *structural molecular activity* (3%) (Fig 5A). Among these molecular functions, *binding*, *catalytic activity*, *transcription regulator activity* and *transporter activity* were classified as the main categories related to the CDDP-resistant phenotype and their principal subcategories are showed in Fig 5B.

### Molecular pathway analysis

**Fig 6:**
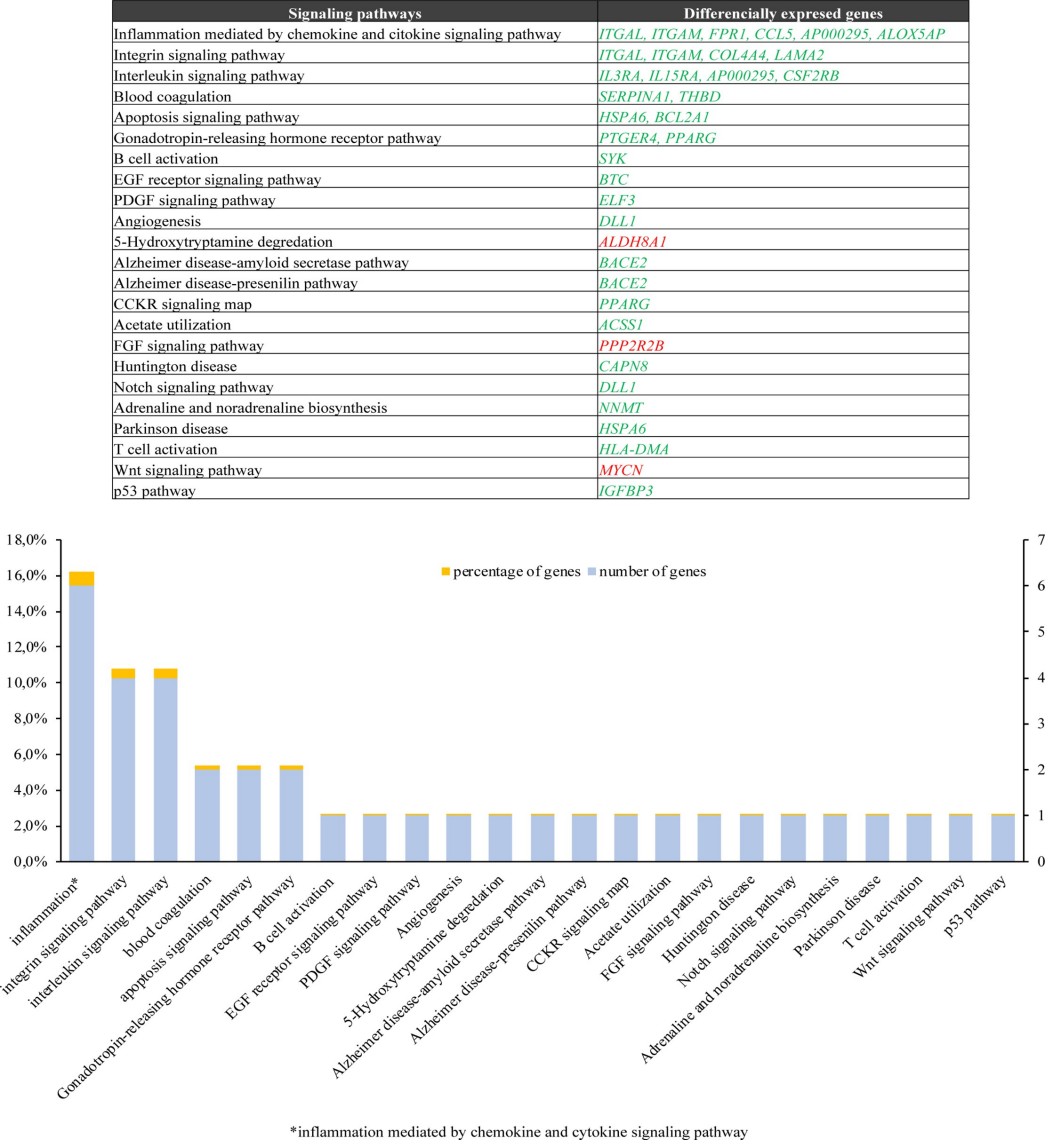
Signaling pathways analysis of DEGs involved in cisplatin-resistant models. Differential expressed genes (DEGs) were subjected to pathways analysis using PANTHER classification system. Up-regulated genes are shown in green and down-regulated genes are shown in red. DEGs were mainly associated to *inflammation mediated by chemokine and cytokine signaling* pathway, *integrin signaling* pathway and *interleukin signaling* pathway.

The signaling pathways analysis demonstrated that DEGs were enriched for 23 metabolic pathways, among which the most enriched were: *inflammation mediated by chemokine and cytokine signaling pathway* (16%), *integrin signaling pathway* (11%) and *interleukin signaling pathway* (11%). Within *inflammation mediated by chemokine and cytokine signaling pathway*, 6 up-regulated genes were enriched; *ITGAL*, *ITGAM*, *FPR1*, *CCL5*, *AP000295* and *ALOX5AP*. Within the integrin signaling pathway, 4 up-regulated genes were enriched: *ITGAL*, *ITGAM*, *COL4A4* and *LAMA2*. Finally, within the interleukin signaling pathway, 4 up-regulated genes were enriched: *IL3RA*, *IL15RA*, *AP000295*, *CSF2RB*. (Fig 6). Likewise, a GO of DEGs was performed using the Metascape tool, obtaining similar results according to the inflammatory process (S1 Fig).

### Validation of RNA-seq analysis in parental and CDDP-resistant GC cell lines

**Fig 7:**
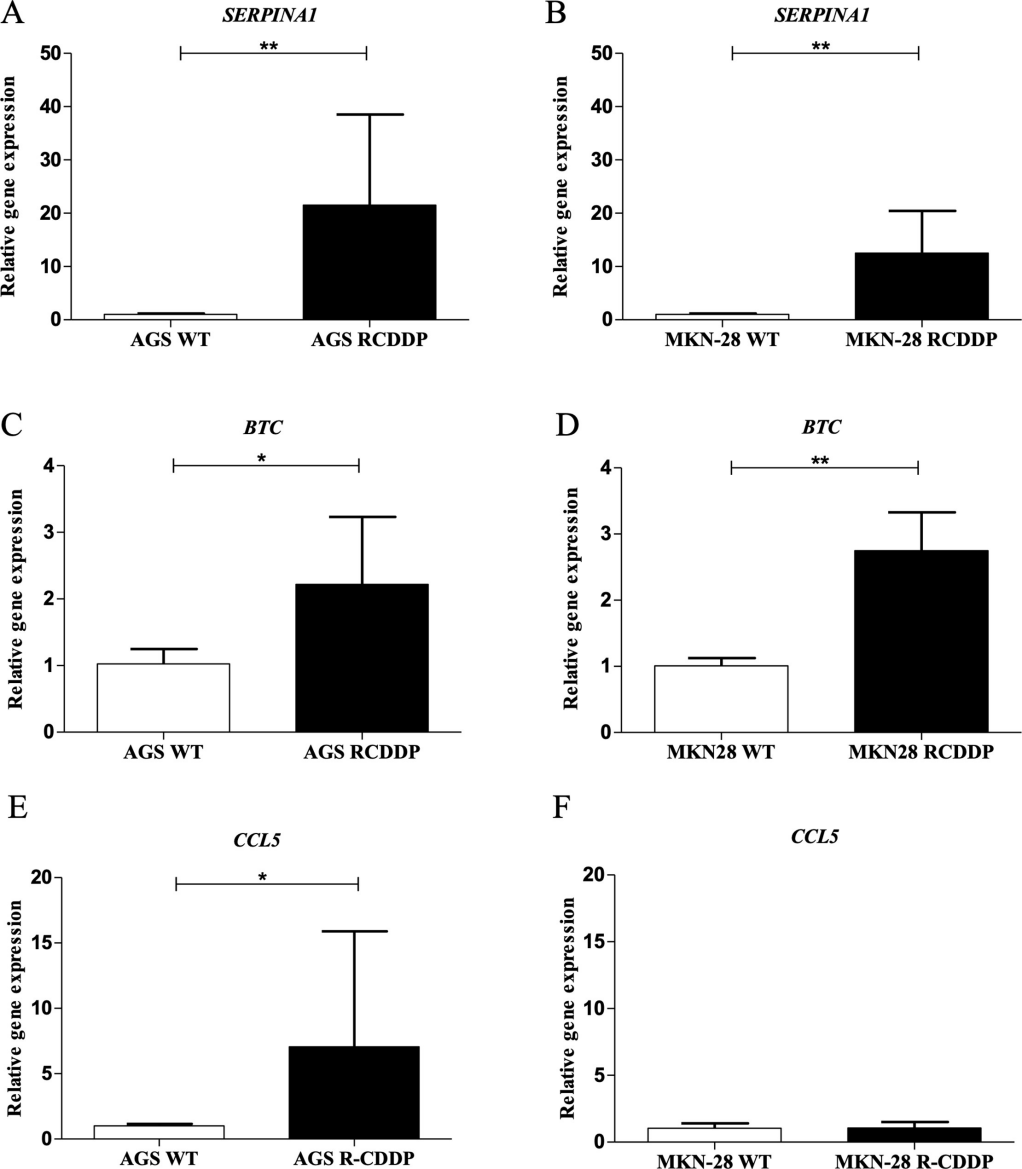
Validation of transcriptomic analyses by qRT-PCR. Three up-regulated genes were identified to be differentially expressed between CDDP-resistant and parental cells in the RNA-seq analysis. (A) *SERPINA1* expression in AGS WT cells and AGS R-CDDP cells; (B) *SERPINA1* expression in MKN-28 WT and MKN-28 R-CDDP cells; (C) *BTC* expression in AGS WT and AGS R-CDDP cells; (D) *BTC* expression in MKN-28 WT and MKN-28 R-CDDP cells. (E) *CCL5* expression in AGS WT cells and AGS R-CDDP cells; (F) *CCL5* expression in MKN-28 WT and MKN-28 R-CDDP cells. The mRNA expression was evaluated by qRT-PCR using 2^-(ΔΔCT)^ method with *ACTB* gene as control. Mann-Whitney test was used to compare groups. Values of P<0.05 were considered statistically significant. *P<0.05, **P<0.01 and ***P<0.001. Data were expressed as mean ± SD of three biological replicates.

**Fig 8:**
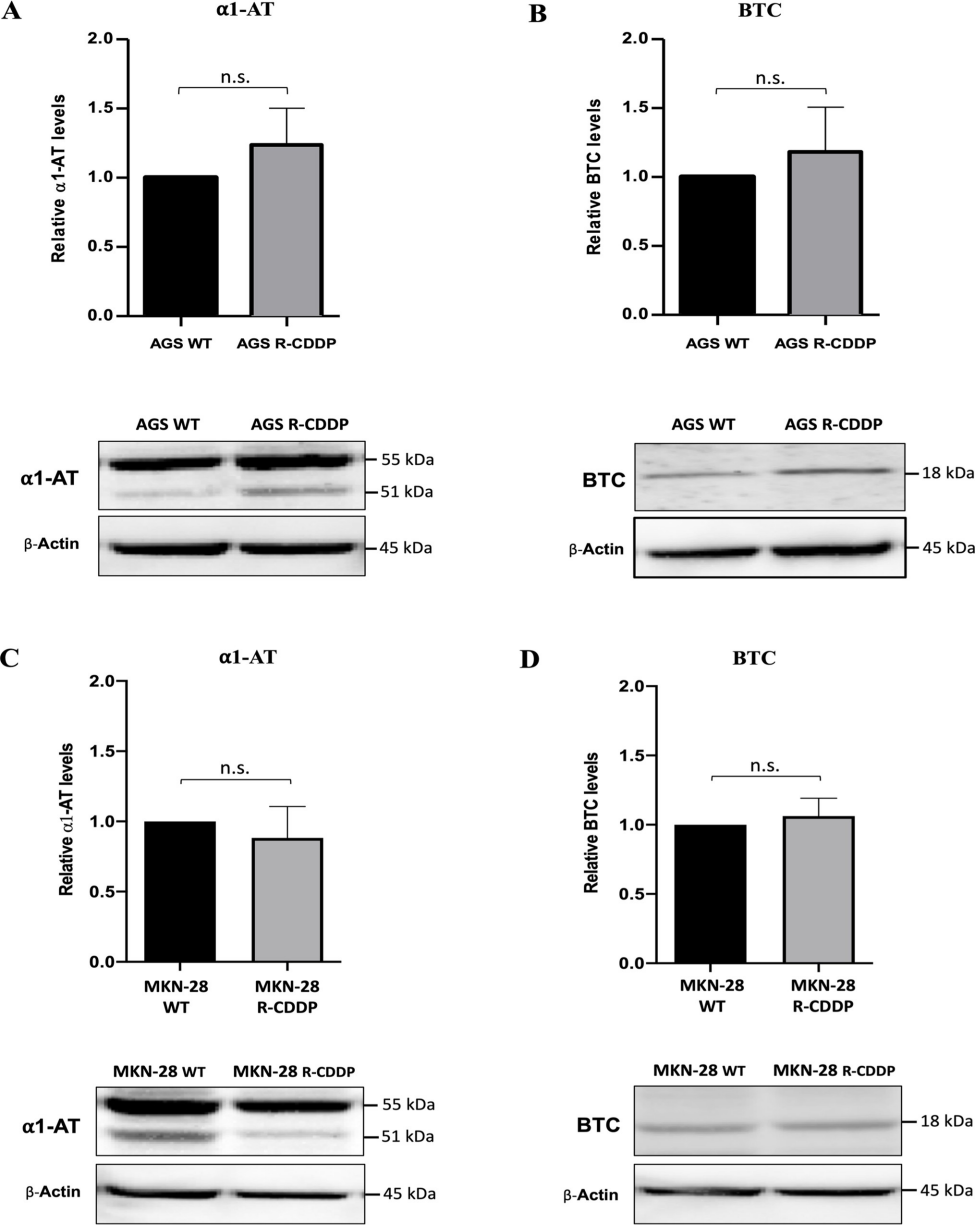
Validation of transcriptomic analyses by western blot. Protein expression levels were analyzed by western blot. Then, densitometry analyses were performed using ImageJ software. (A) α-1-antitrypsin (α1-AT) expression in AGS WT cells and AGS R-CDDP cells; (B) BTC expression in AGS WT cells and AGS R-CDDP cells; (C) α1-AT expression in MKN-28 WT and MKN-28 R-CDDP cells; (D) BTC expression in MKN-28 WT and MKN-28 R-CDDP cells. β-Actin was used as control. The relative expression was quantify using arbitrary units. Mann-Whitney test was used to compare groups. Values of P<0.05 were considered statistically significant. *P<0.05, **P<0.01 and ***P<0.001. Data were expressed as mean ± SD of three biological replicates.

The relative expression of *SERPINA1*, *BTC* and *CCL5*, three up-regulated DEGs that were associated to CDDP resistance in the RNA-seq analysis, was confirmed by qRT-PCR and western blot analyses. Regarding mRNA expression levels, a significant increase in *SERPINA1* mRNA expression was observed in AGS R-CDDP cells (P<0.01; Fig 7A) and MKN-28 R-CDDP cells (P<0.01; Fig 7B) in comparison with their parental cells. Also, a significant increase in *BTC* mRNA expression was observed in AGS R-CDDP cells (P<0.05; Fig 7C) and MKN-28 R-CDDP cells (P<0.01; Fig 7D) in comparison with their parental cells. Moreover, a significant increase in mRNA levels of *CCL5* was observed only in AGS R-CDDP cells (P<0.05 Fig 7E) compared to AGS WT cells, but not in MKN-28 R-CDDP cells in comparison with MKN-28 WT cells (Fig 7F). Regarding protein expression levels, a slight but no-significant increase in protein levels of α-1 antitrypsin (α1-AT; protein encoded by *SERPINA1* gene) and BTC (Fig 8A and 8B, respectively) were shown in AGS R-CDDP cells compared to AGS WT cells. As well, no significant changes were observed in α1-AT and BTC protein levels between MKN-28 R-CDDP cells compared to MKN-28 WT cells (Fig 8C and 8D, respectively). Unfortunately, CCL5 protein expression was not possible to be evaluated in both models in a reliable manner (data not shown).

## Discussion

Platinum chemotherapeutic agents such as CDDP are currently used in the treatment of GC [4]. Unfortunately, chemoresistance is the most important cause of treatment failure and mortality in GC [8]. Much effort has been put into understanding the mechanisms of CDDP chemoresistance; however, the underlying mechanisms are not fully understood. The development of CDDP-resistant models and the identification of DEGs would aid to the understanding the molecular mechanisms underlying chemoresistance as well as the identification of new therapeutic targets for the treatment of GC [13,27]. Different CDDP-resistance models in GC have been previously proposed [28] [29] [30]. Recently, the cell line SGC-7901/DDP was suggested as CDDP-resistance model in GC and its expression profile was characterized using Next-generation sequencing [31]. However, parental SGC-7901 cells -from which the resistant cells were developed–were shown to be actually derivatives from HeLa cell line [32] [33]. Therefore, the development of more reliable model is necessary to assess the molecular mechanisms and DEGs involved in CDDP-resistance.

In the present study, a model for studying resistance to CDDP in GC have been proposed from two initial cell lines: AGS and MKN-28. These cells acquired resistance through a method based on increasing drug doses stepwise for a period of 12 months. In the first part of this study, we characterize each resistance model through cell viability assays, cell death and relative expression of molecular markers involved in CDDP resistance.

As expected, the EC_50_ values and resistance index (RI) of AGS R-CDDP and MKN-28 R-CDDP cells (~5.4-fold and ~6.8-fold change, respectively) confirmed that these cells presented a significantly greater tolerance to higher CDDP concentrations compared to parental counterparts [13]. Although the exact level of CDDP resistance is difficult to define in patients and *in vitro* and *in vivo* studies, a minimum threshold of 2-fold of resistance has been widely accepted in clinical studies because responses have been observed using the double of the standard clinical dose of CDDP in drug-intensive therapy protocols [34]. Another previous studies performed in non-small cell lung cancer reached a RI of ~10-fold for CDDP compared to parental cells [35], which denotes the importance of the different cell types when CDDP resistance is studied.

Cell death assays showed that the CDDP-resistant cell lines AGS R-CDDP and MKN-28 R-CDDP had significantly lower cell death rates than their parental cell lines. These results agree with numerous studies showing that tumor cells resistant to chemotherapy escape cell death to protect against antitumor treatment [36–38]. In this regard, the decrease of cell death can be attributed to alterations in the signaling pathway of apoptosis, such as an increase in anti-apoptotic molecules and/or a decrease or defective function of pro-apoptotic proteins [39].

Regarding relative expression of molecular markers involved in CDDP resistance, three molecular markers were used to characterize each model cell; *ABCC2*, *CTR1* and *ATP7A*. *ABCC2*, member 2 of the ATP-binding cassette (ABC) transporter subfamily C, has been previously implicated in CDDP resistance because is one of the main transporters involved in the efflux of this drug [6,40]. The copper transporter 1 (CTR1), a transmembrane protein involved in copper homeostasis, is a major influx transporter for platinum drugs in multiple cell systems [41,42], and its knockout has been related to the induction of platinum drugs resistance *in vitro* and *in vivo* [41]. On the other hand, *ATP7A*, a copper transporting P-type ATPase, has been also implied in CDDP resistance in cancer [43,44] by inducing the efflux of platinum drugs[45]. In this study, *CTR1* gene was downregulated in AGS R-CDDP and MKN-28 R-CDDP cells, suggesting that *CTR1* might be involved in CDDP resistance in both cell lines by decreasing the influx of this drug. These results were concordant with those obtained in CDDP-resistant A2780 human ovarian carcinoma cell line (A2780cis), which showed decreased CDDP accumulation and lower *CTR1* expression compared to parental A2780 cell line [46]. In addition, the lower levels of *CTR1* mRNA found in ovarian tumors were associated with poor clinical outcome [47]. Only AGS R-CDDP cells showed a significant overexpression of *ABCC2*, which was concordant with the *ABCC2* overexpression related to the drug resistance contribution in mammalian cells [48,49], meanwhile in MKN-28 R-CDDP cells the expression of *ABCC2* was found down-regulated. On the other hand, *ATP7A* showed a significant decrease in MKN-28 R-CDDP cells but not in AGS R-CDDP where non-statistical differences were observed between CDDP-resistant and parental cells. Although higher *ATP7A* levels mediate platinum drugs resistance in different cell lines [50,51], the discrepancies in *ATP7A* expression have been previously found in ovarian tumors prior and after treatment, thus additional studies are needed to validate this gene as a molecular marker of resistance [50,52].

Once the CDDP-resistant cell lines were established, a transcriptome analysis was performed to identify the DEGs between CDDP-resistant cells and parental cells. The RNA-seq analysis showed 189 DEGs of which 139 were involved in molecular functions including *binding*, *catalytic activity*, *molecular transduces activity*, *transcription regulator activity*, *transporter activity*, *molecular function regulator* and *structural molecular activity*. As drug resistance is a multifactorial process, many of these molecular functions such as *binding*, *catalytic activity*, *transcription regulator activity* and *transporter activity* are closely related to the platinum-drug resistant phenotype [53,54].

*Binding* category has been previously described as a principal molecular function in several transcriptomic analyses. Ahn *et al*. performed RNA-seq analysis in melanoma models of resistance: BRAF inhibitor-sensitive A375P BRAF V600E cells, BRAF inhibitor-resistant counterparts (A375P/Mdr), and SK-MEL-2 BRAF-WT cells with intrinsic resistance to BRAF inhibitors. These analyses showed that the most DEGs were enriched in binding category (more than 40% of 5,660 genes) [55]. Similarly, Men *et al*. showed that a high proportion of DEGs found between tamoxifen-sensitive and tamoxifen-resistant MCF-7 breast cancer cell line belonged to the binding subcategories (2,836 genes, representing 93.35% of all DEGs) [56]. Moreover, Fang *et al*. also found a high proportion of DEGs within the binding category (946 genes, representing 89.16% of all DEGs) when CDDP-resistant lung adenocarcinoma cell line A549 was compared to its parental A549 cell line [54]. All these studies are concerned with our work, which the category "binding" was the most enriched molecular function in conjunction with catalytic activity.

*Catalytic activity* was another of the most enriched functions in GO analysis, which also presented the *hydrolase activity* and *oxidoreductase activity* as its main subcategories. When CDDP enters into the cytoplasm is activated by a series of hydrolysis reactions carried out preferentially by thiol-containing molecules, such as glutathione (GSH) and metallothionines [34,57]. An increase of GSH levels has been related with CDDP resistance [58]. CDDP forms conjugates with GSH by glutathione S-transferases (GSTs) and these conjugates can be exported from cells by ATP-dependent glutathione S-conjugate export (GS-X) pump, contributing to resistance phenotype [43,59].

Another of the most enriched molecular functions in the GO analysis was *transcription regulator activity*. Transcription factors contribute to drug-induced responses and can induce either transient or acquired drug resistance [60]. Many transcription factors involved in resistance to CDDP have been differentially expressed in this study, such as *NRA42* (Nuclear Receptor subfamily 4 group A member 2; P<0.001), an orphan nuclear receptor that confers chemoresistance in GC and colorectal cancer [61,62].

On the other hand, as alteration of drug flux (increased efflux and/or decreased influx) is one of the principal mechanisms of drug resistance. Decreased drug accumulation is a consistent feature of many CDDP-resistant cell lines, mainly attributed to membrane transporter proteins [10]. In this regard, GO analysis also showed that many DEGs were associated with *transporter activity* category, particularly with *transmembrane transporter activity*. Membrane transporter proteins such as *ATP7A*, *ATP7B* [63] could contribute to resistance by increasing CDDP efflux or by decreasing CDDP influx in a similar manner of *CTR1* role [41]. In our study, similar targets such as *ATP9A* and *ATP10A* were differentially expressed. Because ATP-dependent efflux protein, is one of the main mechanisms responsible for multi-drug resistance (MDR) [40], these targets could be playing a role in CDDP resistance.

According to our results, three signaling pathways were mainly involved in our CDDP-resistant models: *inflammation mediated by chemokine and cytokine signaling pathway*, *integrin signaling pathway* and *interleukin signaling pathway*. Cytokines are low molecular weight proteins involved in the induction and regulation of interactions of the immune, inflammatory and hematopoietic systems [64,65]. Within the classification of cytokines can be found interleukins and chemokines; the interleukins are secreted by leukocytes and can induce the immune activation on other leukocytes, while the chemokines are involved in several aspects of the behavior of the leukocytes, for example chemotaxis [65]. On the other hand, integrins are responsible for the cellular binding to most of extracellular matrix proteins (e.g. collagens, fibronectin, and laminins) and function as transmembrane anchors between the extracellular matrix and the actin cytoskeleton [66]. In addition, integrins can be expressed in leukocytes (for example integrins β2) to join immunoglobulins and other proteins involved in the inflammatory process [65]. Interestingly, some of these DEGs (e.g. integrins *ITGAL* and *ITGAM*) are involved in two signaling pathway, such as *inflammation mediated by chemokine and cytokine signaling pathway* and *integrin signaling pathway*. The interleukin receptor *AP000295* has been found to participate in both *the inflammation mediated by chemokine and cytokine signaling pathway* and *interleukin signaling pathway*. Therefore, this background not only suggest that the resistant cell lines have been undergoing a pro-inflammatory process but also that CDDP resistance can be enhanced by these pro-inflammatory mechanisms.

Several studies have focused their attention on the regulation of multi-drug resistance during an inflammatory response, in particular by pro-inflammatory cytokines [67]. Cytokine expression active a variety of signaling pathways involved in drug resistance, either by autocrine regulation (cytokines secreted by tumor cells) or by paracrine regulation (cytokines secreted by stromal cells or tumor-associated cells; e.g. fibroblasts), resulting in a differential expression in drug resistant and sensitive cell lines (e.g. overexpression of interleukin-6 (*IL6)* and interleukin-8 (*IL8)* in resistant cell lines) [68]. Evidences suggest that the chemokines, cytokines and growth factors secreted by cancer-associated fibroblasts (CAFs), a cell subpopulation present in tumor microenvironment, facilitate the drug resistance development in gastric cancer [69]. In this context, a recent study showed that *IL-6* secreted by CAFs is a critical contributor to chemoresistance in GC cells through the activation of the Jak1-STAT3 signaling pathway [70]. On the other hand, other inflammatory mediators such as *NFκB* (Nuclear Factor Kappa B) and *TNF* (Tumor Necrosis Factor) have also been associated to drug resistance [71]. Despite the evidence about the crosstalk between components of the immune system and cancer cells can influence chemoresistance, more studies are needed for a better understanding of these resistant mechanisms [71].

Finally, the higher mRNA expression of *SERPINA1* (α-1-antitrypsin), *BTC* (Probetacellulin) and *CCL5* (C-C motif chemokine ligand 5), three up-regulated genes associated to CDDP resistance according to RNA-seq analysis, was confirmed in CDDP-resistant cells compared to their parental counterparts. *SERPINA1* and *BTC* belong to the top five differential expressed genes associated to CDDP resistance, meanwhile that *CCL5* was involved in the most enriched metabolic pathway; *inflammation mediated by chemokine and cytokine signaling pathway*.

GO analysis confirmed that *SERPINA1*, *BTC* and *CCL5* are involved in molecular functions related to drug resistance. *SERPINA1* was involved in molecular functions such as *binding*, *catalytic activity* and *molecular function regulator*; *BTC* was implied in molecular functions including *binding*, *molecular transducer activity* and *molecular function regulator*, meanwhile *CCL5* was also involved in all the molecular functions described above.

According to PANTHER Classification System, the protein encoded by *SERPINA1* is a serine protease inhibitor involved in endopeptidase inhibitor activity, protease binding and serine-type endopeptidase activity. Higher levels of *SERPINA1* has been associated with chemoresistance of human epithelial ovarian cancer [72] and breast cancer [73] and has also been proposed as a potential serum biomarker for GC [74]. However, the role of *SERPINA1* in resistance to CDDP in GC has not been clarified yet.

On the other hand, *BTC* is a member of epidermal growth factor (EGF) family involved in cell proliferation, epidermal factor receptor binding and positive regulation of cell proliferation [25]. High expression of *BTC* has been previously associated with worse survival in GC [75]; however, there are no studies that relate *BTC* gene with CDDP resistance in GC cancers.

Besides, *CCL5* also known as Regulated upon Activation, Normal T-cell Expressed and Secreted (RANTES), is a chemokine secreted in a paracrine or autocrine fashion which has been associated with enhance the cancer progression in multiple myeloma (MM), classical Hodgkin lymphoma (cHL), prostate, breast, gastric, colon, and ovarian cancer, and melanoma [76]. During the last decade, *CCL5* has been shown to be an inductor of resistance to taxane [77], CDDP [78], tamoxifen [79] and Src inhibitors [80] in models of prostate cancer, ovarian cancer and breast cancer, respectively. However, the *CCL5* role in the development of chemoresistance for any of the drugs commonly used for GC has not been evaluated.

In this work, *SERPINA1* and *BTC* showed a differential expression of mRNA profile between parental and CDDP-resistant cells, as shown in the literature. Meanwhile *CCL5*, only showed a differential expression of mRNA profile between parental and CDDP-resistant AGS cells, which can be explained for the differences in molecular background between AGS[14] and MKN-28[15] cells.

Regarding protein levels, no differences were observed for α-1-antitrypsin (α1-AT; protein encoded by *SERPINA1*), meanwhile CCL5 protein could not be detected reliably by western blot analysis. According The Human Protein Atlas [81], α1-AT can be located intracellularly or by secreting, meanwhile CCL5 protein on the membrane or secreting. This is important because our western blot analyses were performed using only cellular pellets, therefore, is probably that levels of α1-AT and CCL5 protein should be detected using the supernatant. ELISA and chemokine arrays are methods that have been used to confirm protein levels quantitative or semi-quantitative screening, respectively. In the particular case of tumor cells, the influence of CCL2 chemokine on gastric cancer has been studied with a preliminary screening using chemokine array validated with ELISA in the supernatant of the cell culture, indicating that CCL2 is being secreted, as we infer to happen with CCL5, which would explain the negative results of western blot [82]. In addition, CCL2 and CCL5 have been detected by cytokine array in the supernatant of stromal cells promoting the resistant phonotype of ovarian cancer cell lines [83]. In addition, *SERPINA1* and *CCL5* can undergo post-transcriptional regulation related with miRNA, like mir-940 [84] and miR-214 [76], respectively, which could explain the no correlation between mRNA and protein levels. Others no-correlation factors between mRNA and proteins levels are summarized in Maier *et al* [85].

Similarly, no significant differences were observed for BTC in AGS R-CDDP and MKN-28 R-CDDP compared to their wild-type counterparts. These results can be also explained by post-transcriptional mechanisms[86] [87]. However, these factors appear to be less frequent in the resistance developed in the AGS cell line, where at least a tendency towards an increase in α1-AT and BTC proteins was observed.

Since this is an initial screening to study of molecular mechanisms involved in resistance to CDDP in GC, functional assays such as knockdown or overexpressing of genes involved in CDDP-resistance should be performed to determine the effect on CDDP sensitivity, as well as, the results obtained from cell lines must be validated in clinical samples for future studies.

## Conclusions

Characterization studies have effectively demonstrated that AGS R-CDDP and MKN-28 R-CDDP are reliable models of CDDP resistance because resemble the resistant phenotype of GC and show a significant higher tolerance to increasing concentrations of CDDP. These features were confirmed by significant differences in the RNA expression of known resistance markers (*CTR1* gene) and new molecular markers of CDDP resistance (*SERPINA1*, *BTC* and *CCL5*).

GO analysis showed that *binding*, *catalytic activity*, *transcription regulator activity* and *transporter activity* were found among the four most enriched molecular functions in these models, which have been related to CDDP resistance in previous studies. In addition, several DEGs found in RNA-seq were mainly associated to pathways related to inflammation mediated by chemokine and cytokine that could induce many of the CDDP-resistant phenotype in GC and could be used as potential therapeutic targets for the treatment of gastric cancers resistant to CDDP.

This is the first study where a transcriptomic analysis was performed in a reliable *in vitro* model of CDDP-resistant gastric cancer. However, cancer patient samples and other complementary analyses are needed for future studies in order to clarify the role of signaling pathways associated with inflammation process in gastric cancers resistant to CDDP and achieve a more clinical utility of these findings.

## Supporting information

S1 Fig Gene ontology (GO) analysis of DEGs in RNA-seq analysis by Metascape tool. A) Metascape enrichment clustering analysis shows the P-value statistical significance among the different GO terms. B) Genes involved in three principal signaling pathways.
(TIFF)
