# Supplementary figures and images for: Functional and transcriptomic characterization of cisplatin-resistant AGS and MKN-28 gastric cancer cell lines
Source: PLoS One. 2020 Jan 28;15(1):e0228331. doi: 10.1371/journal.pone.0228331 (PMC6986722; doi:10.1371/journal.pone.0228331)

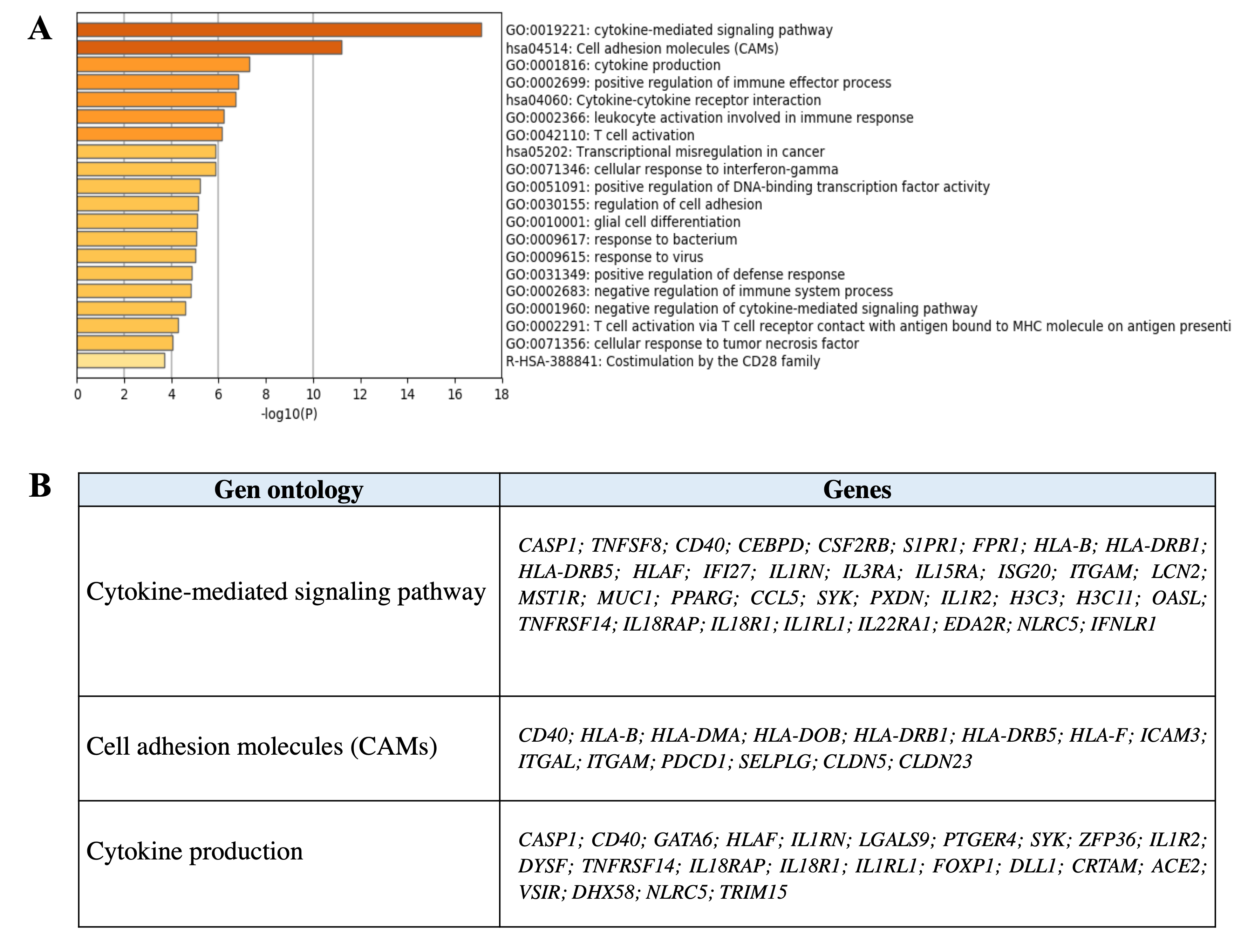

Supplement: S1 Fig — A) Metascape enrichment clustering analysis shows the P-value statistical significance among the different GO terms. B) Genes involved in three principal signaling pathways. (TIFF) [file pone.0228331.s001.tiff]
